# Applying LASSO logistic regression for the prediction of biliary complications after ex vivo liver resection and autotransplantation in patients with end-stage hepatic alveolar echinococcosis

**DOI:** 10.1186/s40001-024-01898-1

**Published:** 2024-05-29

**Authors:** Xin Lin, Ying-Mei Shao, Rui-Qing Zhang, Tuerganaili Aji

**Affiliations:** 1 Centre of Digestive and Vascular Surgery, The First Affiliated Hospital of Xinjiang Medical University, Urumqi, 830000 Xinjiang China; 2 State Key Laboratory of Pathogenesis, Prevention and Treatment of High Incidence Diseases in Central Asia, Urumqi, 830000 Xinjiang China

**Keywords:** Hepatic alveolar echinococcosis, Ex vivo liver resection and autotransplantation, Liver transplantation, Hepatectomy, Biliary complications

## Abstract

**Background:**

The purpose of this study was to explore the relevant risk factors associated with biliary complications (BCs) in patients with end-stage hepatic alveolar echinococcosis (HAE) following ex vivo liver resection and autotransplantation (ELRA) and to establish and visualize a nomogram model.

**Methods:**

This study retrospectively analysed patients with end-stage HAE who received ELRA treatment at the First Affiliated Hospital of Xinjiang Medical University between August 1, 2010 and May 10, 2023. The least absolute shrinkage and selection operator (LASSO) regression model was applied to optimize the feature variables for predicting the incidence of BCs following ELRA. Multivariate logistic regression analysis was used to develop a prognostic model by incorporating the selected feature variables from the LASSO regression model. The predictive ability, discrimination, consistency with the actual risk, and clinical utility of the candidate prediction model were evaluated using receiver operating characteristic (ROC) curves, calibration plots, and decision curve analysis (DCA). Internal validation was performed by the bootstrapping method.

**Results:**

The candidate prediction nomogram included predictors such as age, hepatic bile duct dilation, portal hypertension, and regular resection based on hepatic segments. The model demonstrated good discrimination ability and a satisfactory calibration curve, with an area under the ROC curve (AUC) of 0.818 (95% CI 0.7417–0.8958). According to DCA, this prediction model can predict the risk of BCs occurrence within a probability threshold range of 9% to 85% to achieve clinical net benefit.

**Conclusions:**

A prognostic nomogram with good discriminative ability and high accuracy was developed and validated to predict BCs after ELRA in patients with end-stage HAE.

## Highlights

- This study used the LASSO logistic regression model to establish a nomogram for predicting biliary complications after ex vivo liver resection and autotransplantation.
- This study is one of the studies with the largest number of ELRA patients included thus far.
- The four common clinical features included in our model can be easily applied in the clinic.

## Introduction

Hepatic alveolar echinococcosis (HAE) is a fatal parasitic infection caused by Echinococcus multilocularis larvae [1, 2]. The majority of cases, approximately 91% worldwide, are concentrated in Northwest China [2–4]. In cases where the HAE has not invaded crucial vascular structures, the main treatment option is a combination of albendazole and radical surgical resection [5, 6]. Unfortunately, due to the growth characteristics of HAE, including its insidious onset, long incubation period, and invasive tumor-like growth, 40% of patients are diagnosed after HAE has invaded crucial intrahepatic vascular structures, such as the portal vein, hepatic vein, inferior vena cava, and left and right hepatic ducts [6–9]. However, radical surgery often provides limited benefits for end-stage HAE patients [7, 10]. Historically, liver transplantation has been an effective treatment for end-stage HAE. Nevertheless, due to the limited availability of organs, high surgical costs, and the lifelong negative impact of immunosuppressive drugs, liver transplantation is unfeasible for everyone.

In 1988, Pichlmayr first applied ELRA to patients with previously inoperable, complex, space-occupying liver lesions. This new approach offers a promising solution for complex intrahepatic lesions [11]. Unfortunately, its efficacy in the treatment of liver malignancies does not appear to be sufficient [12–14]. Subsequent studies demonstrated that ELRA produced promising outcomes in the treatment of complicated non-cancerous liver masses. In 2018, Aji et al. [6] conducted a clinical investigation of ELRA that included 69 patients with end-stage HAE. The median postoperative follow-up was 22.5 months (14–89 months). This study revealed that ELRA is a safe, feasible, and effective treatment for end-stage HAE, with no recurrence observed after the procedure. ELRA is also superior to in situ liver transplantation, because it does not require a liver donor or lifelong immunosuppressive drugs. However, the complexity of this surgical procedure increases the susceptibility of ELRA to postoperative complications, including a notable incidence of BCs. These complications can have a significant impact on patient quality of life and overall survival.

Postoperative complications of ELRA include ascites, postoperative bleeding, infection, and biliary complications [6, 15–17]. However, there is limited research on the risk factors for major postoperative complications of ELRA, particularly biliary complications. To address these gaps, we analysed a series of patients with end-stage HAE treated with ELRA. This study aimed to establish a nomogram model to evaluate patients who underwent ELRA to help clinicians predict the risk of biliary complications and provide a more personalized treatment strategy.

## Materials and methods

### Ethics

This retrospective study with anonymous data was approved by the Ethics Committee of the First Affiliated Hospital of Xinjiang Medical University (K202312-42/20211015–53) and conducted in accordance with the Declaration of Helsinki. Informed consent was not required, because the study was retrospective, and the data were anonymized.

### Data sources

Clinical data were collected from 159 end-stage HAE patients who underwent ELRA surgery at the First Affiliated Hospital of Xinjiang Medical University between August 1, 2010, and May 10, 2023. After rigorous screening, 118 patients were included and divided into two groups based on the presence or absence of biliary complications (BCs) as the outcome event: the BC group (*n =* 48) and the non-BC group (*n =* 70).

### Data collection

Patient demographic data, preoperative laboratory tests, imaging results, intraoperative details, and postoperative laboratory tests and imaging results were collected.

### Inclusion and exclusion criteria

Inclusion criteria:

1. The age range was 18–65 years.
2. The confirmation of HAE requires preoperative imaging data and postoperative pathological results.
3. Clinical data were complete or had less than 20% missing data.
4. Postoperative complications of patients were definitively diagnosed by clinicians or through image evaluation.

Exclusion criteria:

1. Patients aged outside of 18–65 years.
2. Patients with ambiguous pathological results or the absence of HAE.
3. Patients with 20% or more incomplete clinical data.

## Preoperative, intraoperative and postoperative details of ELRA

### Preoperative evaluation

Preoperative computed tomography (CT) and magnetic resonance imaging (MRI) of the liver were used to objectively evaluate the dimensions and location of the lesion, the extent of hepatic invasion, the degree of vascular and biliary involvement, and the presence of extrahepatic metastases.

Liver volume, lesion volume and remnant liver volume (RLV) were calculated using a three-dimensional (3D) imaging analysis system. The calculation method of standard liver volume (SLV) was based on the definitionscalculated according to the methods of Tongyoo et al. [18] and Urata et al. [19].

### Indications for ELRA

The diagnostic criteria for HAE followed the World Health Organization (WHO) guidelines [20]. Based on “Ex vivo liver resection and autotransplantation” by Aji et al., the indications for ELRA in advanced HAE patients can be summarized as follows:

1. All three hepatic veins are involved simultaneously.
2. The secondary or superior branches of the portal vein are involved bilaterally.
3. The confluence of the inferior vena cava is involved.
4. In situ curative resection may not be feasible.

In addition, patients must be in good physical condition with relatively normal hepatic and renal function, with total bilirubin less than twice the upper limit of normal, and any hepatic echinococcosis lesions must be controlled with albendazole or completely resectable by surgery. In addition, it is recommended that the ratio of estimated residual liver volume (RLV) to standard liver volume (SLV) be greater than 0.4 and that the patient have a Child‒Pugh score of at least B, with an indocyanine green retention rate at 15 min (ICG R15) of less than 15%.

### ELRA procedure

The ELRA surgical procedure can be broadly divided into four components [6]. The first stage involves simultaneous resection of the entire liver and surrounding invaded organs or tissues. The second step involves temporary reconstruction of major blood vessels to maintain haemodynamic stability during the anhepatic phase. The third component involves the precise removal of HAE lesions while the organ is perfused with organ preservation solution at 0–4 °C. In addition, the major vessels and bile ducts of the remnant liver were repaired. The fourth step involves reimplantation of the autograft via vascular reconstruction. Intraoperative records included details of combined resections, transfusion volumes, duration of each surgical step, actual RLV measurements and timing of inferior vena cava (IVC) and portal vein (PV) occlusion.

### Postoperative treatment and follow-up

After surgery, all patients were transferred to the intensive care unit (ICU) for monitoring and treatment. This includes antimicrobial therapy, acid suppression, volume resuscitation, and maintenance of internal environmental stability. Once their condition has stabilized, they are transferred to specialized wards. Postoperative monitoring typically involves blood biochemistry, abdominal ultrasound, and CT scans to monitor the volume of the remnant liver and blood flow in the portal vein and inferior vena cava. This helps to detect and manage postoperative complications early.

According to the literature recommendations, all subjects should receive oral albendazole therapy for 2 years after discharge [21]. Regular postoperative follow-up, such as an abdominal CT scan every 3–6 months, is essential to identify potential complications and evaluate the probability of recurrence.

### Data analysis

The statistical analyses for this study were conducted using R software (version 4.2.2, http://www.R-project.org). *P* values were calculated using two-tailed tests with a significance level of *P* < 0.05. Variables with a missing data rate higher than 20% were excluded from the analysis. Multiple imputation methods were used to fill in the remaining missing data. LASSO regression was employed to identify the most significant variables. Multivariate logistic regression analysis was subsequently conducted to validate the selected predictive factors and establish a predictive model based on the results of the LASSO regression analysis. A nomogram model was created to estimate an individual’s risk of biliary complications after ELRA. Receiver operating characteristic (ROC) curves were used to evaluate the model's predictive performance, and the area under the curve (AUC) was calculated. We performed 1000 bootstrap resamplings to construct a calibration curve and assess the agreement between the predicted and actual values. Decision curve analysis (DCA) was used to evaluate the clinical utility of the model by calculating the net benefit across different risk threshold probabilities.

## Results

### Patient characteristics

A total of 118 patients, comprising 49 males and 69 females, were included in this study after screening. The BC group had 48 patients, while the NBC group had 70 patients. Table 1 displays the clinical variables and perioperative characteristics of the participants, which revealed significant differences in age, hepatic bile duct dilation, portal hypertension, splenomegaly, and regular resection based on hepatic segments (*P* < 0.05).

**Table 1:** Baseline characteristics of patients

Clinical characteristic variables	NBC (*N* = 70)	BC (*N* = 48)	p
Sex			0.355
Male	32 (45.7%)	17 (35.4%)	
Female	38 (54.3%)	31 (64.6%)	
Age	35.5 ± 11.2	40.3 ± 10.6	0.023
BMI, kg/m^2^	21.4 ± 2.8	22.3 ± 3.9	0.183
PSH			0.314
No	37 (52.9%)	20 (41.7%)	
Yes	33 (47.1%)	28 (58.3%)	
PTCD			0.353
No	56 (80%)	34 (70.8%)	
Yes	14 (20%)	14 (29.2%)	
TB, μmol/L	31.0 ± 42.0	31.3 ± 34.2	0.965
ALT, U/L	67.4 ± 71.5	74.3 ± 111.4	0.705
AST, U/L	87.7 ± 146.6	84.3 ± 132.9	0.896
ALB, g/L	34.8 ± 5.4	36.7 ± 8.5	0.175
GGT, U/L	159.7 ± 161.0	176.8 ± 219.4	0.644
Child‒Pugh			0.96
A	67 (95.7%)	45 (93.8%)	
B	3 (4.3%)	3 (6.2%)	
Extrahepatic invasion			0.362
No	45 (64.3%)	26 (54.2%)	
Yes	25 (35.7%)	22 (45.8%)	
Combined resection			0.782
No	51 (72.9%)	33 (68.8%)	
Yes	19 (27.1%)	15 (31.2%)	
portal hypertension			0.004
No	61 (87.1%)	30 (62.5%)	
Yes	9 (12.9%)	18 (37.5%)	
Splenomegaly			0.037
No	57 (81.4%)	30 (62.5%)	
Yes	13 (18.6%)	18 (37.5%)	
Hepatic bile duct dilation			< 0.001
No	43 (61.4%)	9 (18.8%)	
Yes	27 (38.6%)	39 (81.2%)	
MaxD, cm			0.49
< 10	52 (74.3%)	32 (66.7%)	
≥ 10	18 (25.7%)	16 (33.3%)	
Anhepatic phase, min			0.055
< 486.5	63 (90.0%)	37 (77.1%)	
≥ 486.5	7 (9.5%)	11 (22.9%)	
Bloodloss, ml			0.106
< 1150	41 (58.6%)	20 (41.7%)	
≥ 1150	29 (41.4%)	28 (58.3%)	
Blood transfusion, U	5.6 ± 5.5	6.8 ± 5.6	0.24
Postoperative biliary drainage			0.877
No	43 (61.4%)	31 (64.6%)	
Yes	27 (38.6%)	17 (35.4%)	
Regular resection			0.043
No	57 (81.4%)	46 (95.8%)	
Yes	13 (18.6%)	2 (4.2%)	
Biliary reconstruction			0.589
B1	22 (31.4%)	11 (22.9%)	
B1	47 (67.1%)	36 (75%)	
B3	1 (1.4%)	1 (2.1%)	
Number of biliary anastomoses			0.829
1	56 (80%)	40 (83.3%)	
2	14 (20%)	8 (16.7%)	
RV/SLV, %	74.1 ± 24.8	74.2 ± 19.9	0.978
GRWR, %	1.5 ± 0.5	1.4 ± 0.4	0.714
POTB, μmol/L	65.2 ± 72.4	76.0 ± 62.8	0.402
POALT, U/L	138.2 ± 153.0	105.9 ± 87.6	0.15
POAST, U/L	92.0 ± 223.9	47.1 ± 29.3	0.102
POALB, g/L	38.7 ± 5.4	37.4 ± 4.8	0.168
POGGT, U/L	133.2 ± 191.0	129.8 ± 102.2	0.899
POALP, U/L	170.8 ± 347.1	142.9 ± 94.3	0.524

This table shows the baseline characteristics

BMI, body mass index; PSH, preoperative surgical history; PTCD, percutaneous liver puncture biliary drainage; TB, total bilirubin; ALT, alanine aminotransferase; AST, aspartate transaminase; ALB, albumin; GGT, glutamyltranspeptidase; MaxD, maximum diameter of lesion; B1, bile duct end-to-end anastomosis; B2, bile duct jejunal anastomosis; B3, B1 + B2; RV/SLV, residual liver volume/standard liver volume; GRWR, graft receptor body weight ratio; POTB, postoperative total bilirubin; POALT, postoperative alanine aminotransferase; POAST, postoperative aspartate transaminase; POALB, postoperative albumin; POGGT, postoperative glutamyltranspeptidase; POALP, postoperative alkaline phosphatase

### Feature variable selection

**Fig. 1:**
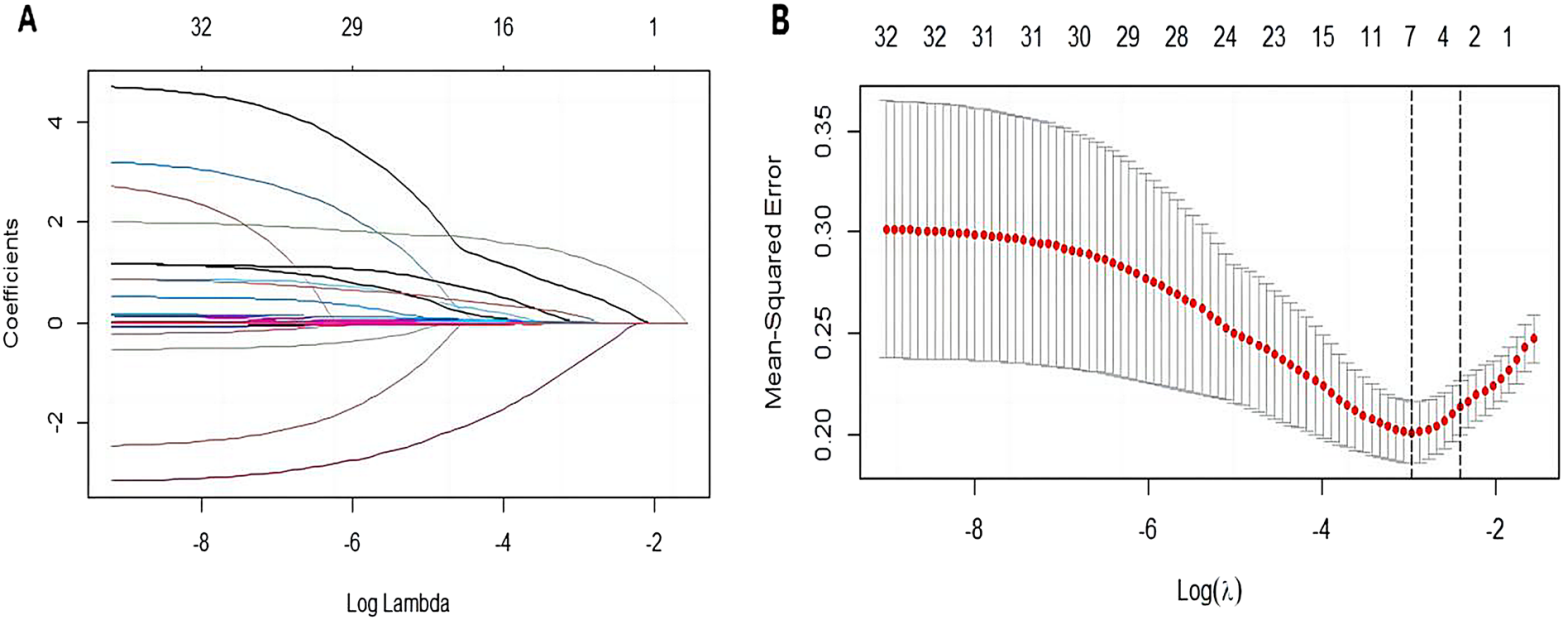
Selection of the optimum clinical feature variables based on the LASSO binary logistic regression model. **A** 32 included feature variables were screened and a coefficient profile plot was generated to show the log (lambda) sequence. **B** Minimum lambda value was determined using tenfold cross-validation. LASSO regression identified four independent variables with nonzero coefficients when the minimum lambda was 0.051

The LASSO regression analysis identified 32 variables, three of which had nonzero coefficients and were considered potential risk factors: age, hepatic bile duct dilation, and portal hypertension. Furthermore, regular resection of hepatic segments was identified as a potential protective factor (Fig. 1A, B).

### Multivariate logistic regression analysis

The results of the multivariate logistic regression analysis showed that age, hepatic bile duct dilatation, and portal hypertension were independent risk factors for biliary complications after ELRA. Furthermore, regular resection based on hepatic segments was identified as a protective factor (Table 2).

**Table 2:** Risk factors for multivariate analysis

Variable	Multivariate analysis		
	β-Coefficient	OR(95%CI)	*P*
Age	0.043	1.04(1–1.09)	0.035
ALB	NA	NA(NA)	NA
BL	NA	NA(NA)	NA
BMI	NA	NA(NA)	NA
HBDD	1.904	6.71(2.62–17.19)	< 0.001
PH	1.288	3.62(1.22–10.76)	0.02
RR	– 2.031	0.13(0.02–0.75)	0.022

ALB, albumin; BL, blood loss; BMI: body mass index; HBDD, Hepatic bile duct dilation; PH, portal hypertension; RR, Regular resection based on hepatic segments

### Establish and visualize a personalized predictive model

**Fig. 2:**
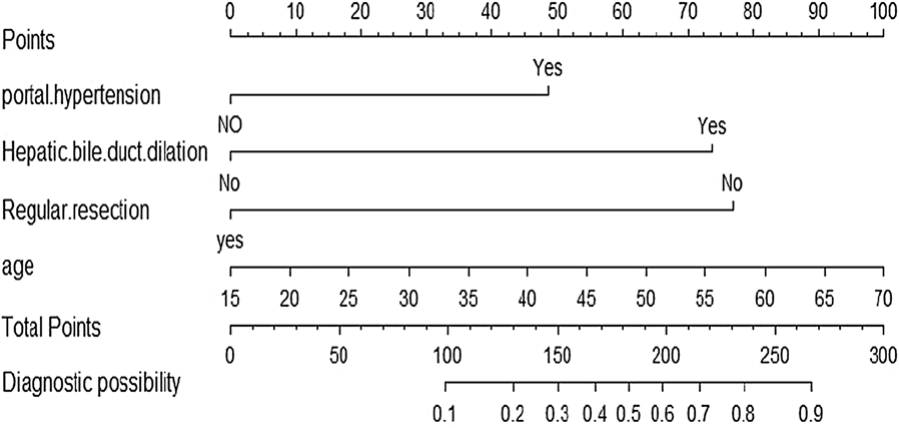
Nomogram for predicting biliary complications. The nomogram assesses the probability of biliary complications on a scale from 0 to 300. For each predictive factor, draw a vertical line on the evaluation axis and record the corresponding points. Add the points from each predictor to obtain the total score, which corresponds to the predicted probability of major postoperative complications at the bottom of the nomogram

Based on logistic regression analysis, a nomogram model was developed to predict risk factors for BCs after ELRA (Fig. 2).

### Evaluation and verification of nomogram

**Fig. 3:**
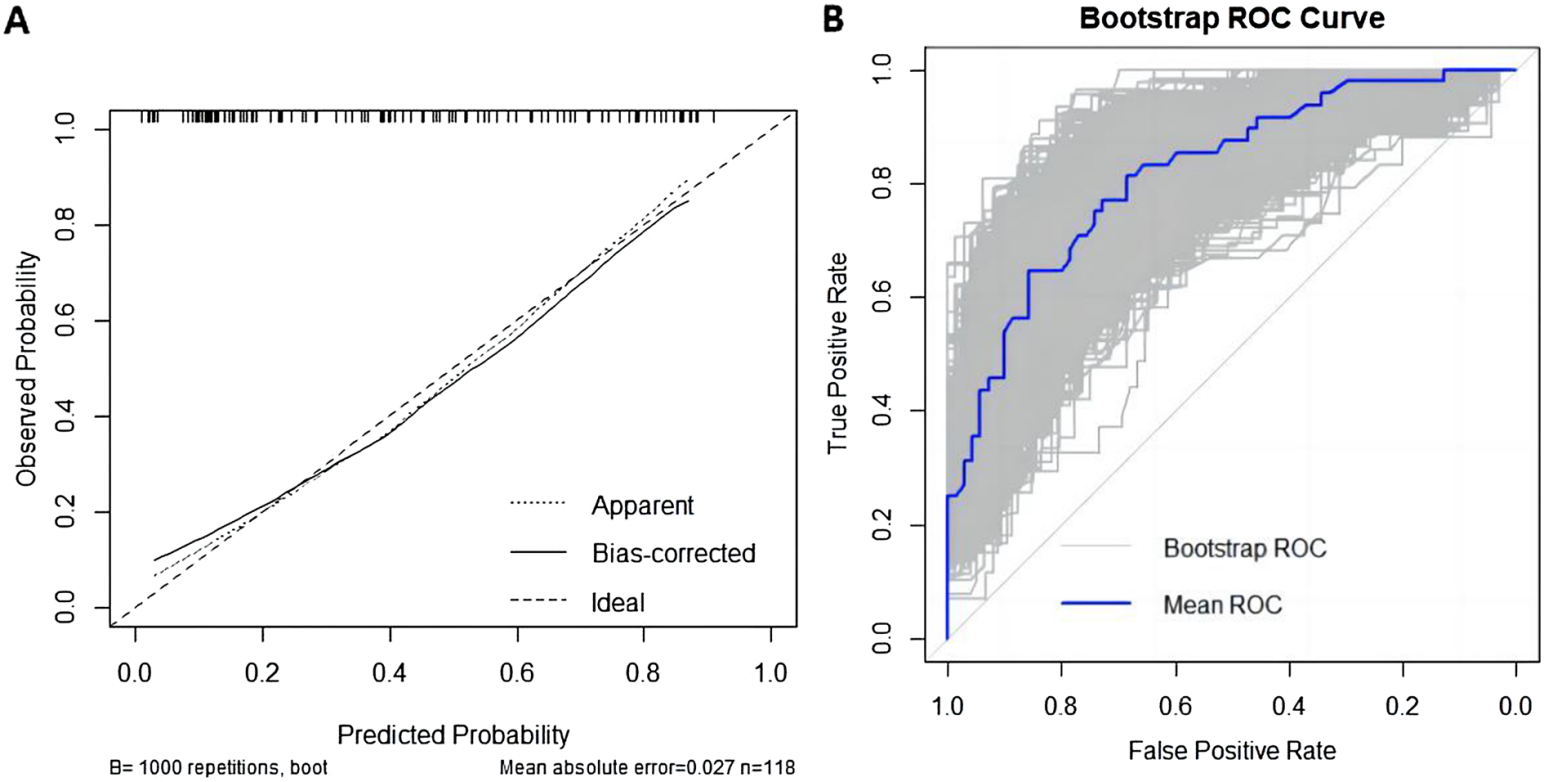
Calibration curves and receiver operating characteristic (ROC) curve analysis for BCs after ELRA. **A** Calibration curves of the nomogram model. The *x*-axis represents the predicted risk of BCs. The *y*-axis represents the actual diagnosis. **B** Receiver operating characteristic (ROC) curve analysis for BCs after ELRA.AUC = 0.818(95% CI 0.7417–0.8958)

**Fig. 4:**
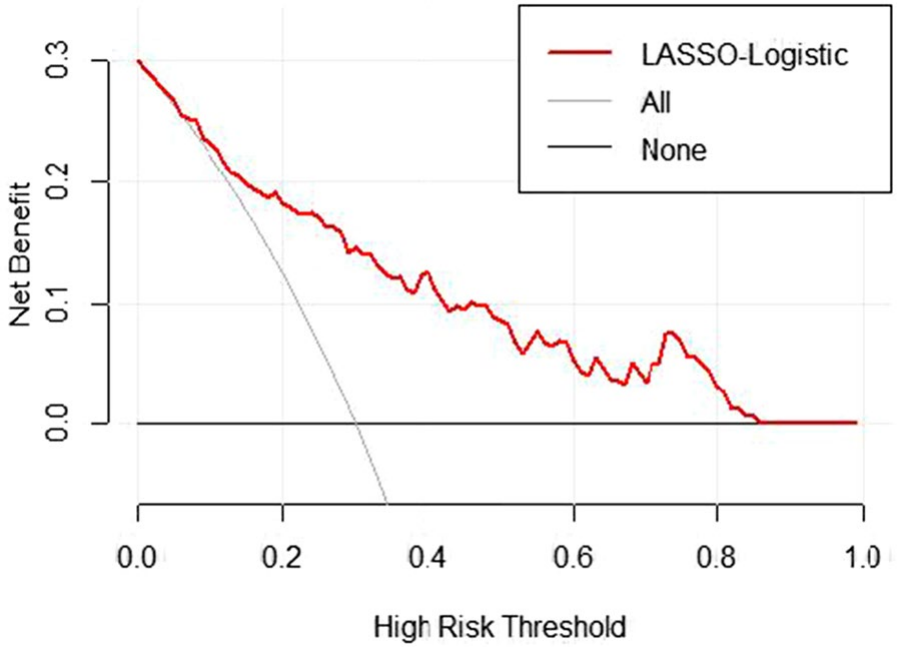
Decision curve analysis (DCA) of the nomogram prediction. When the threshold probability is > 9% and < 85%, using this predictive model to identify BCs after ELRA could provide a net clinical benefit

The bootstrap sampling method (resampling: 1000) was used for internal validation to evaluate the predictive performance of the developed model for postoperative complications after ELRA. The receiver operating characteristic (ROC) curve (Fig. 3A) demonstrated an area under the ROC curve (AUROC) of 0.818 (95% CI 0.7417–0.8958), indicating satisfactory predictive performance. The calibration curve, presented in Fig. 3B, demonstrates a high level of agreement between the predicted probabilities and actual observations, with no indication of overfitting. To further evaluate the models, clinical decision curve analysis (DCA) was performed, as shown in Fig. 4. The study results suggest that the patient benefit curve (red curve) performed better than the two alternative curves (the black horizontal line 'None' indicates no intervention, and the gray diagonal line 'All' indicates no differential intervention) between the ranges of 9–85%.

## Discussion

In 1988, Pichlmayr’s team proposed the concept of extracorporeal liver surgery based on their experience with hepatectomy and liver transplantation. This approach offers a novel method for managing complex intrahepatic space-occupying lesions [11]. For complex intrahepatic space-occupying lesions, ELRA has two advantages: it eliminates the need to wait for a suitable liver donor, as required in donation after cardiac death (DCD) or living donor liver transplantation (LDLT), thereby sparing patients from the potential side effects of lifelong immunosuppressive therapy [22]. Second, ELRA provides superior exposure of the lesion and ample operative space, facilitating precise and thorough resection of complex intrahepatic lesions compared to in vivo resection [23]. These distinct advantages position ELRA as a valuable option for treating challenging intrahepatic lesions.

In this study, 48 out of 118 patients (40.68%) experienced biliary complications after ELRA, which is higher than the reported incidence of biliary complications following orthotopic liver transplantation (5–30%) [24]. When comparing the outcomes of liver transplantation from donation after circulatory death (DCD) and living donors, it is important to consider the impact of end-stage hepatic artery embolization (HAE) on the patient's liver. During surgery, complex biliary tract reconstruction may be necessary due to factors such as invasion of important blood vessels and bile ducts, preoperative residual cavity infection, prolonged jaundice, and biliary obstruction. These factors can lead to a greater incidence of postoperative biliary complications in ELRA patients.

After ELRA, biliary complications can be classified into two types: biliary leakage and biliary obstruction. In our study, 23 out of 118 patients (19.49%) experienced bile leakage, while 37 out of 118 patients (31.36%) experienced biliary obstruction. It is important to note that some patients experienced both bile leakage and biliary obstruction, which is why the total number of affected patients did not match the statistical results. This study revealed that age, preoperative hepatobiliary dilatation, and portal hypertension were independent risk factors for biliary complications after ELRA. Furthermore, regular hepatectomy based on liver segments was identified as a protective factor. This discussion will primarily focus on these characteristics.

Previous studies on orthotopic liver transplantation have suggested that biliary complications are not age dependent [25]. However, more recent research indicates a correlation between the age of the donor and the occurrence of biliary complications after liver transplantation. For instance, Foley et al. [26] reported that the incidence of postoperative biliary complications significantly increased when the donor was over 40 years old.

Biliary complications may be related to hepatocyte regeneration. When hepatocytes are injured, they activate S-phase- and mitosis-specific genes. However, older livers have fewer hepatocytes entering the S-phase, and the speed at which hepatocytes enter this phase is slower, which affects the speed of liver regeneration [27]. Additionally, the telomere length of hepatocytes decreases with age. Before the age of 40, telomere shortening in hepatocytes appears to be more significant when cells are rapidly renewed and growing [28]. Furthermore, aging inhibits regeneration of hepatocytes at the epigenetic level and reduces their responsiveness to epidermal growth factor (EGF). These factors may slow liver regeneration and increase the incidence of biliary complications [27–30].

In addition, it has been observed that elderly patients may have a decreased tolerance to ischaemia‒reperfusion injury in their liver histiocytes. Experimental data have demonstrated that I/R injury can significantly affect the biliary tree. This type of injury primarily occurs during the reperfusion period and is caused by cold ischemia, nutrient deprivation, and the accumulation of metabolic byproducts [31]. Liver ischaemia‒reperfusion injury involves complex biological processes such as anaerobic metabolism, oxidative stress, calcium overload, and cytokine release from Kupffer cells (KCs) and neutrophils [32]. The prolonged anhepatic period during ELRA leads to tissue hypoxia, metabolic derangements, and cholangiocyte and hepatocyte death due to inadequate hepatic blood supply [33]. This finding is in line with our results and clarifies the reason for the lower mean age of the nonbiliary complication (NBC) group (34.5 ± 11.8) compared to that of the biliary complication (BC) group (40.3 ± 10.6), which was significantly different (*p* = 0.023).

**Fig. 5:**
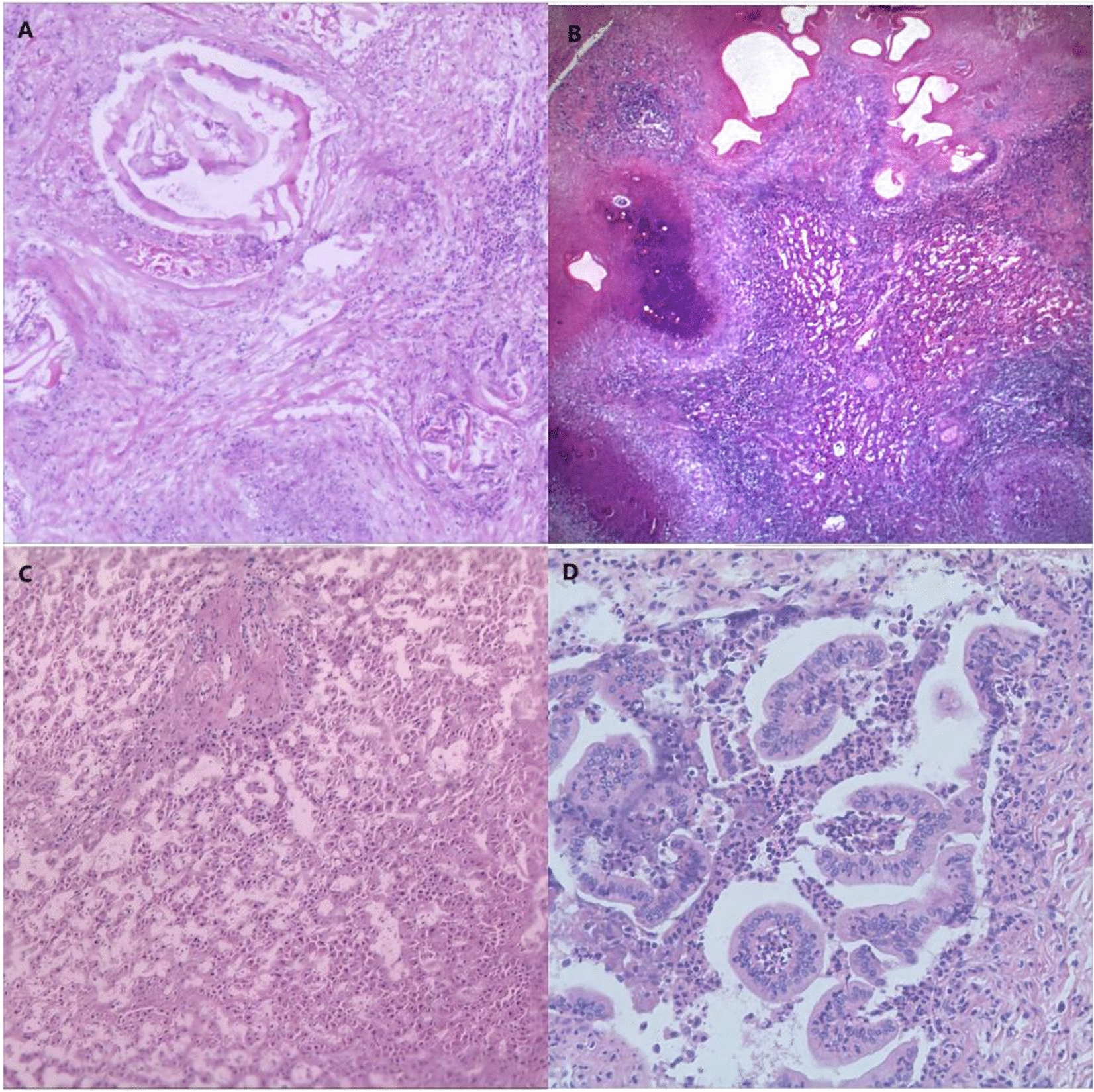
Postoperative pathological results of the lesion and liver tissue obtained from HAE patients who underwent ELRA surgery. **A** Dilation of the hepatic duct. The cut end of the bile duct shows invasion by HAE. **B** HAE with necrosis, brownish-yellow pigment deposition, calcification, and multinucleated giant cell reaction. **C** Intrahepatic cholestasis and bile duct obstruction occur within the small bile ducts. **D** Dilation of the intrahepatic bile ducts with partial detachment of the biliary epithelium accompanied by lymphocytic infiltration, microabscess formation, and cholestasis. Lymphocytic infiltration and fibrosis in the hepatic hilum

Preoperative HAE lesions that invade the biliary tract at the first porta hepatis and cause biliary obstruction often result in hepatobiliary dilatation. This can lead to complications such as jaundice and infection, which can damage biliary tissue cells. Yoshidome et al. [34] reported that obstructive jaundice can cause damage to the sinusoidal endothelial cells of the bile duct. A liver with obstructive jaundice may be more vulnerable to ischemia/reperfusion injury than a normal liver, and this effect may have long-lasting consequences. This susceptibility is not limited to the damage caused by long-term high bilirubin levels on biliary endothelial cells. Furthermore, the toxic effects of bilirubin may impact the healing of liver wounds and the growth of biliary anastomoses after ELRA, potentially affecting other organs in patients. In addition, biliary complications may be attributed to infections caused by HAE. The healing of bile ducts after surgery is affected by the regeneration of bile duct endothelial cells and the formation of small scars resulting from preoperative inflammation of the bile ducts [35]. Pathological findings of dilated bile ducts in HAE patients after ELRA also revealed fibrous tissue proliferation (Fig. 5).

Portal hypertension is a clinical condition that occurs when there is obstruction and/or increased blood flow in the portal vein system, leading to a sustained increase in hydrostatic pressure in the portal vein and its branches. The underlying mechanism involves several factors, such as extrahepatic portal vein obstruction, compression of the first hepatic portal vein, cirrhosis, and hepatic outflow tract obstruction.

In patients with end-stage HAE, portal hypertension is mainly caused by invasion of the first hepatic portal vein and/or lesion invasion of the second hepatic portal outflow tract. Portal hypertension has been identified as a risk factor for biliary complications after ELRA, which may be related to changes in the biliary tree induced by portal hypertension. As early as 1944 and 1965, Fraser et al. [36] and Williams et al. [37] reported associations between jaundice and common bile duct compression with portal hypertension. Biliary changes are commonly observed on imaging studies in patients with portal hypertension, although the exact pathogenesis remains unclear. Currently, most researchers widely accept that the mechanism of bile duct compression and ischemia is portal hypertension, which leads to obstruction of the bile duct venous plexus and the venous plexus around the bile duct. This results in bile duct congestion and compression [38, 39]. Prolonged poor blood flow in the bile duct contributes to the formation of arteriolar microthrombi, which further causes damage to bile duct capillaries and endothelial cells [35]. This can impair wound and anastomotic healing and increase the risk of biliary complications.

ELRA may cause minor bile leakage from surgical wounds or from small bile ducts that are missed during the procedure. To reduce the risk of bile leakage, regular hepatectomy based on liver segments may be performed to avoid bile duct damage and prevent intraoperative omission of small bile ducts without ligation, as long as sufficient residual liver quality is ensured. Prior to autologous transplantation, white gauze can be placed on the liver section to check for the presence of bile, or a diluted methylene blue solution can be injected through the cystic duct. A blue wound indicates that the wound is leaking. To close the leak, absorbable sutures or titanium clips can be used. Intraoperative bile duct exploration through the cystic duct is an effective method for detecting bile duct leakage. This method not only preserves the integrity of the common bile duct but is also easy to perform [40]. In addition, while ensuring the remnant liver volume, regular resection of the remnant liver, which involves resection along the remaining liver segments, can effectively reduce the wound area and lower the risk of biliary complications [41]. To ensure the remnant liver volume, a preoperative quantitative assessment of the liver is necessary. This involves precise measurements of the multidimensional target lesion boundary as well as the structural function of the liver and biliary system. Quantitative assessment entails multidimensional and precise measurements of the boundaries of the target lesion, as well as the structure and function of the liver and biliary system. This assessment involves quantifying liver reserve function, accurately measuring liver volume, and precisely delineating the extent of lesions and pathological boundaries. This study provides a foundation for making decisions regarding quantitative liver resection and designing surgical procedures.

The Child‒Pugh scoring system and the indocyanine green (ICG) clearance test are commonly used to evaluate liver function reserve and measure the functional volume of the liver. This provides a quantitative basis for determining the safe limits of liver resection. The safety limit of liver resection (SLLR) refers to the maximum resection volume allowed for an individual while preserving only the essential functional liver volume (EFLV). The calculation of the SLLR relies on the amount of functional liver volume that remains in the patient's healthy liver. The quantification of ICG can serve as a useful reference indicator for determining the SLLR.

In LDLT, there are reports that the GRWR can be reduced to 0.6%, with no significant difference in survival rates compared to a GRWR > 0.8%. Currently, ELRA borrows the evaluation standards for the required graft volume in living donor liver transplantation (LDLT). In LDLT, there are reports that the GRWR can be reduced to 0.6%, with no significant difference in survival rates compared to GRWR > 0.8% [42]. However, it is important to note that a low GRWR is associated with a worse prognosis [43]. In general, a graft-to-recipient ratio (GRWR) ≥ 0.8% is considered the safety limit [44–46].

Currently, there is no conclusive evidence linking the type of biliary anastomosis to the occurrence of biliary complications after ELRA. In this study, the incidence of biliary complications was lower with end-to-end biliary anastomosis (9.32%) than with biliary-enteric anastomosis (30.51%) (*P* = 0.307). However, the method of anastomosis depends on the location of the lesion invading the liver segments and the health of the biliary tract of the graft. Nevertheless, considering that patients with end-to-end anastomosis have normal sphincter function, which is more in line with the natural physiological state of the human body and can effectively reduce the stimulation of intestinal content reflux on the biliary tract, end-to-end anastomosis of the biliary tract can theoretically reduce the incidence of postoperative biliary complications.

The anhepatic phase has been considered a potential risk factor for major postoperative complications in several studies. However, our study did not provide direct statistical evidence to support this claim. Nevertheless, our results indicated a trend towards a prolonged anhepatic phase and an increased incidence of postoperative biliary complications. Further validation with a larger sample size is necessary to confirm these findings.

## Conclusion

Age, hepatic bile duct dilation and portal hypertension are independent risk factors for BCs after ELRA. Under the premise of ensuring the safety limit of liver resection, intraoperative regular resection based on hepatic segments can reduce postoperative biliary complications. For patients at high risk of such complications, appropriate interventions based on the nomogram should be performed.

## Limitations

This study has several limitations: (1) As a prediction model, the sample size included in this study was relatively insufficient; (2) This was a retrospective study, and there was selection bias and other confounding factors when patients were included. The time span is long, the duration of ELRA surgery is constantly improving, and the initial patient status may affect the clinical results; and (3) No multicenter samples were included, and there was a lack of external samples for validation. In the future, we will expand the sample size and perform multicenter joint research to further verify our model. In conclusion, we believe that the model established in this study is helpful for evaluating the occurrence of BCs after ELRA and can provide guidance for clinical decision-making.

## Data Availability

The data used in this study were obtained from the case records of patients treated at the First Affiliated Hospital of Xinjiang Medical University. The datasets generated and analysed during the current study are available from the corresponding author or first author upon reasonable request.
